# Comparison of Apical Sealing Ability of Lateral Condensation Technique in Room and Body- Simulated Temperatures (An in vitro study)

**Published:** 2013-03

**Authors:** F Sobhnamayan, S Sahebi, F Moazami, M Borhanhaghighi

**Affiliations:** a Dept. of Endodontics, School of Dentistry, Shiraz University of Medical Sciences, Shiraz, Iran; b General Dentist

**Keywords:** Body Simulated Temperature, Room Temperature, Lateral Condensation, Dye Penetration

## Abstract

**Statement of Problem:** Studies reported that nearly 60% of endodontic failures have been attributed to inadequate obturation of the root canal system. Thus, complete obturation of the root canal system and proper apical seal are essential elements in the long-term success of root canal treatment.

**Purpose:** This study aimed to compare the apical seal of lateral condensation technique in the room and in body- simulated temperature.

**Materials and Method:** In this experimental study, 70 extracted, single- rooted, human premolar teeth were instrumented and divided up into four groups. All tooth’s canals were obturated by lateral condensation technique except the teeth in the positive control group. Group 1and 2, each with 30 teeth, were obturated in the room and intracanal temperature respectively. The other two groups were positive and negative control group each with 5 teeth. All groups except negative control were covered by two layers of nail polish. Then linear dye penetration was evaluated with a stereomicroscope. Data was analyzed with student-t test and also Kolmogorov- Smirnov Goodness- of- Fit test to make sure of data.

**Results:** Results showed that dye penetration in group one (obturation in room temperature) was 0.6mm more than group 2 (obturation in simulated-body temperature) although this was not statistically significant (*p*> 0.05).

**Conclusion**: Under the condition of this invitro study, apical sealing ability was better in the body-simulated temperature than the room temperature, although it was not statistically significant.

## Introduction

The complete obturation of root canal systems, and the fluid tight seal is the main goal of endodontic treatment to obtain a complete seal along the entire length of root canal systems [1]. It has been reported that nearly 60% of endodontic failures have been attributed to inadequate obturation of root canal systems [2] and were induced by bacterial microleakage, penetration of substances from apical tissue or even coronal microleakage [3-4]. Because satisfactory canal preparation could affect the obturation quality, different techniques of obturation and preparation are recommended to have well-sealed obturation [5]. Gutta-percha is the most widely used obturating material for its good characteristics such as biocompatibility, inertness, plasticity when warmed, ease of handling and removal in retreating cases [6-7]. Gutta percha is present in two different crystalline forms, alpha and beta. Although there is nearly no difference in mechanical properties of the two crystalline forms, some volumetric changes are associated with temperature changes in Gutta percha [8].

Lateral condensation technique of Gutta-percha is the most popular method and this technique is still trained in most dental schools [9-11]. Although this method is considered as the standard routine, it has been criticized for its lack of homogeneity, poor adaptation to dentinal walls and the possibility of root fracture [6]. It is reported that this technique results in irregularities in the gutta percha mass and may not seal the canal fin and isthmus [12].

To achieve a better 3- dimensional filling of spaces, different warm gutta percha have been developed that employ the gutta percha thermoplasticity. The use of heated gutta percha improves the homogeneity of the filling mass and adaptation to dentinal wall [1, 13]. 

Many evaluations of sealing ability of different obturation techniques (injectable thermoplasticized, warm and lateral) have been carried out [5, 14-16]. All these in vitro studies have been done in the room temperature (25c) where as intracanal temperature is nearly 12ºC more than the room temperature which might change the result of these studies. It is possible the higher temperature of intracanal may perhaps change the plasticity of gutta percha and its sealing ability. 

This study evaluates the sealing ability of lateral condensation technique in two different temperatures, room temperature and intracanal (body simulated) temperature. 

## Materials and Method

**Figure 1a:**
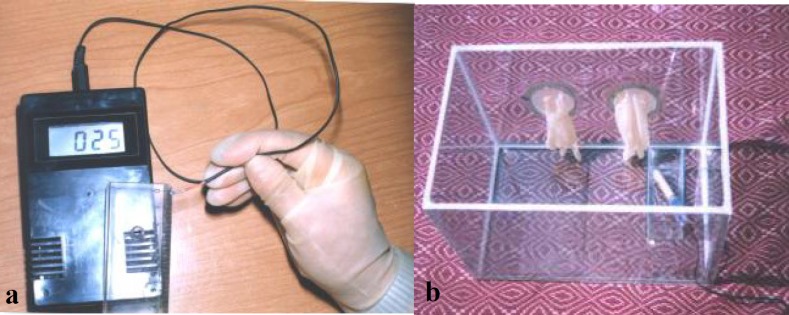
Digital thermometer **b **Glass cavity

In this experimental study a very fine sensor, connecting to a digital thermometer (Nova-Tech, USA), was designed to measure intracanal temperature. This device is operated with alternate current and a 9 volt battery and shows the temperature between 6-500˚c (Figure 1a). 

For evaluation of internal root canal temperature, multiple patients, requisite for root canal therapy were chosen. After cleaning and shaping, each canal was dried with sterile paper points and the disinfected probe was introduced into the canal 3-4 mm beneath the orifice. The temperature in different canals ranged between 33-35˚c. 

Samples were seventy extracted human mandibular premolars. They all had a single and a straight canal with complete apices. Teeth with different anomalies in crowns or roots with severe curvature, crazes or visible fractures were removed from the study. Radiographs were taken bucco- lingually and mesio- distally to identify whether the samples had a direct and single canal. 

The crowns were removed so that the remaining lengths of the roots were about 14-20mm. Soft tissues and calculus were removed from the root surfaces mechanically with hand curettes. All roots were immersed in 0.01% sodium hypochlorite concentration for 24 hours. The working length was recorded with ≠10 or ≠15 k file (Maillefer, Ballaigues, Switzerland). The file was introduced into the canal till its tip came out in the apical foramen, and working length was established 0.5mm shorter that the apex. 

All root canals were prepared with S_1_ to F_2_ protaper NiTi instrument (Dentsply, Maillefer, Ballaigues, S-witzerland)_. _Irrigation with 2ml of 2.5% sodium hypochlorite was performed between the applications of each two files. After preparation teeth were divided into four groups randomly. Two experimental groups (30 teeth in each group). The two experimental groups were obturated with cold lateral condensation technique in two different temperatures with ZOE sealer (Kemdent, UK). 

**Group 1**

Obturation in room temperature (25^o^c): After root canal preparation, canals were dried with sterile paper points. Master gutta-percha was selected for each root canal. The tug back was confirmed in the working length and the proper spreader was selected. ZOE sealer (Kemdent, UK) was prepared according to the factory instructions. Accessory cones were used until they could not be introduced into the canal with more than 2-3mm of their length. Gutta- percha was removed up to 2 mm apical to the orifice with a flame- heated excavator and were cervically sealed with Coltosol (Cavisol, Golchai, Iran). Specimens were radiographed to confirm the competence of root canal obturation and were kept between two wet gauzes.

**Group** **2**

Lateral condensation in intracanal temperature (33˚c- 35˚c): For simulating intracanal temperature two- layer glass cavity (60×40×40cm) was designed with automatic thermostat and completely humidified (100%) atmosphere (Figure 1b). To confirm the temperature, we used the probe connected to the thermometer in the box and checked the temperature.

Teeth in group#2 were transferred into the cavity but gutta- percha, sealer and paper points were kept out of the cavity. After root canal preparation, teeth were transferred into the glass box. Canals were dried with sterile paper points. Master gutta-percha was selected for each root canal. The tug back was confirmed in working length and the proper spreader was chosen. ZOE sealer (Kemdent, UK) was prepared according to the factory instructions in the room temperature. Accessory cones were used until they could not be inserted into the canal more than 2-3mm of their length. Gutta -percha was removed up to 2 mm apical to the orifice with a flame- heated excavator and cervically sealed with Coltosol (Cavisol, Golchai, Iran). Radiographs were taken to check and confirm the obturation quality.

**3- Positive Control**

The similar preparation method was used for this group. Teeth were dried with sterile paper points and one gutta- percha without any sealer were inserted into each canal freely. Coltosol (Cavisol, Golchai, Iran) was used to seal 2-3 mm coronal part of each canal. After taking radiographs, all specimens were preserved at room temperature and in humidified atmosphere. All specimens in the negative control group were prepared and obturated as the group one and sealed with 2-3 mm of Coltosol in the coronal part. 

After canal obturation, all the samples were evaluated regarding the quality of obturation by taking radiographs. Experimental groups and the positive control group were put in 100% humidity and 37ºc for 3 days in the incubator. The apical part of negative control group was coated with a layer of sticky wax in order to prevent dye penetration. Total root surfaces were covered by two layers of nail polish. 

Following obturation root surfaces of experimental groups and positive control group were coated with two layers of nail polish up to the apical 2mm. Then all the teeth were put in room temperature for 72 hours in pelican ink (pelican, Hanover, Germany) in the vertical position so that 3mm of the crown was out of ink. 

The roots were rinsed with tap water for 1 minute and then the nail polish was completely removed with acetone and cotton wool balls. The root surfaces were then evaluated by Zeiss stereomicroscope (Zeiss, Munich, Germany) at × 6 magnification for fracture. 

In order to evaluate dye penetration in the teeth, teeth were cleared by the Tagger method [17]. 

Maximum linear dye penetration was observed under a stereomicroscope (Zeiss, Munich, Germany) at× 6 magnification with an accuracy of 0.25 mm and by three different observers. For each tooth maximum linear dye penetration was recorded and measured by a special ruler with 0.1 mm accuracy. Finally collected data was compared using student T-test and also Kolmogorov- Smirnov Goodness of fit test to make sure of the data. 

## Results

**Figure 2a:**
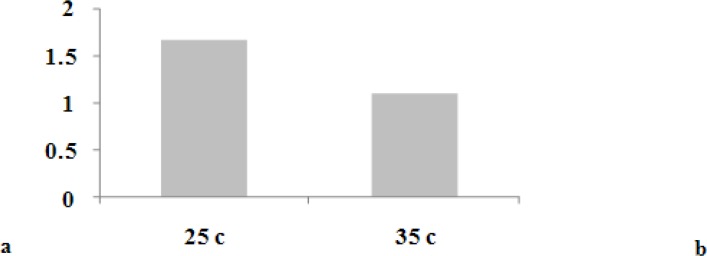
Comparison of microleakage of lateral condensation technique in the room and body-simulated temperature (Mean±SD microleakage, T-Test result) **b** Comparison of microleakage of lateral condensation technique in the room and body-simulated temperature (Mean±SD microleakage Kolmogorov- Smirnov Goodness-of-fit

The negative control group demonstrated no dye penetration while the positive control showed dye penetration along the entire root length. The mean linear dye leakages for the two groups are shown in Table 1, and Figures 2a and b. 

**Table 1:** Mean difference = 0.5848≈0.6

**Number**		**Mean**	**Standard** **Deviation**	**SE of** **Mean**	**P**
Group 1	29	1.6790	0.868	0.347	0.129
Group 2	30	1.0942	0.809	0.148	

**Figure 3a:**
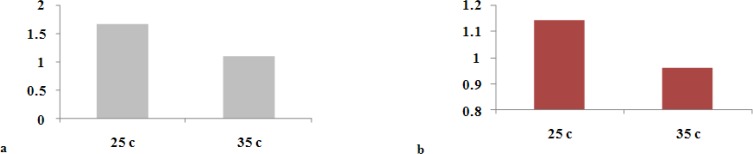
Negative control group **b** Positive control group

T test results showed no significant statistical difference between two groups in dye penetration (*p*> 0.05) although there is a difference between written results. Mean dye leakage of group one with 29 specimens (one tooth was omitted during the procedure) was 1.67 mm and this was 1.09 mm for group 2. The difference was nearly 0.6 mm (Figure 3).

Another statistical test was done by deleting specimens with highest and lowest dye penetration. The difference was not statistically significant despite the mean leakage was 1.14 in 26 specimens in group one and 0.96 in 28 specimens in group two (*p*> 0.05) (Table 2, Figure 2a and 2b).

**Table 2:** Mean of differences = 0.1841

**Number**		**Mean**	**Standard** **Deviation**	**SE of the mean**	**p**
Group 1	26	1.1452	0.804	0.158	0.354
Group 2	28	0.9610	0.646	0.122	

We do also Wilcoxon test in order to evaluate inter observer agreements in data (Table 3). Three observers had agreement on reading the dye microleakage. Kolmogorov- Smirnov Goodness- of- fit test was done to assure the normality of data and results.

**Table 3:** Inter observer agreement

	**A**	**B**	**C**
A	1.0000 (59) *p*=.	0.9643 (59) *p*= 000	r= 0.9246 (59) *p*= 000
B	0.9643 (59) *p*= 000	0.9192 (59) *p*=.	0.9192 (59) *p*= 000
C	0.9246 (59) *p*= 000	0.9192 (59) *p*= 000	1.0000 (59) *p*=.

## Discussion

Apical sealing ability is one of the important criteria of a three-dimensional obturation of the root canal system. Microleakage is a good indicator to assess the sealing ability of root end filling materials. A variety of techniques have been developed to evaluate the microleakage including fluid filtration [18], cross section and microscopic evaluation [19], penetration of trace agents like radioisotopes, dyes, and bacteria and their endotoxins [20-22]. Some of these methods are more reproducible such as fluid filtration and dye extraction techniques [23-26]. The dye penetration test is the most popular technique, because it is easy to perform, it is inexpensive, and it has a high degree of staining [27]. Low weight molecules of dye can penetrate better than bacterial cells [28]. On the other hand, low-molecular-weight dyes are finer than those in the oral cavity or those macromolecular materials which are used in clinical studies [29] the limitation of dye leakage studies is that they can show the leakage only in one plane, making it impossible to evaluate the total microleakage in other planes [13, 30].

We used dye penetration technique using a stereo microscope to evaluate the micro leakage of specimens like many other studies [5, 31-37].

Temperature is an important element that can cause different changes in the gutta percha characteristics. It seems that high temperature makes gutta-percha softer and affects its sealing ability. This study was designed to compare the sealing ability of gutta- percha in the room and body- simulated temperature. In this study, the samples filled in the body-simulated temperature showed less microleakage although not significant. Many of studies in this field are evaluating the sealing ability of the different techniques with and without sealers in room temperature [3-5, 38-40].

Most studies have found that injection- molded thermoplasticized techniques yield apical seals better or similar to the lateral condensation techniques [1, 41-42]. 

Sadeghi et al found no significant difference in the density and sealing ability of lateral compaction using two different spreaders and vertical compaction using a BeeFill device [40]. 

Dadresanfar et al, in 2010, compared the sealing ability of lateral condensation technique and BeeFill system and found that the dye leakage in the BeeFill system was less than lateral condensation technique, although this wasn’t statistically significant [5].

Another study in 2010, showed the better sealing ability of thermoplasticized gutta percha compaction in comparison with the lateral condensation technique, although it was not statistically significant either [4].

The results of these studies are in accordance with the results of our study. However, these and other studies have not evaluated the sealing ability of gutta-percha in body simulated temperature [3, 38-39].

This study showed better penetration of spreader in the group two (35˚c) and subsequently, more accessory cones were placed in each tooth in this group. Results showed that dye penetration, in group 2, was nearly 0.6 mm lesser than group1; although this was not statistically significant. The absence of any significant statistical difference can be explained in this way that this 10-12˚c difference in temperature could not affect the sealing ability of gutta percha in the lateral condensation technique. Thus it seems that the results of the previous studies, regarding the lateral condensation technique, can be extended to invivo situations. Evidently, more studies are needed to evaluate different kinds of obturation techniques in body-simulated temperature. For a better appraisal, other studies can be designed, with more samples and different techniques, to consider these results more reliable.

## Conclusion

Under the condition of this invitro study, apical sealing ability was better in the body-simulated temperature compared to the room temperature, even though it was not statistically significant.
